# Simultaneous Hydatid Cyst of the Liver and Left Iliac Fossa: An Unusual Case Report

**DOI:** 10.1155/2019/9101425

**Published:** 2019-09-03

**Authors:** Tika Ram Bhandari, Sudha Shahi

**Affiliations:** ^1^Department of General Surgery, People's Dental College and Hospital, Kathmandu, Nepal; ^2^Formerly Department of General Surgery, Universal College of Medical Sciences, Bhairahawa, Nepal; ^3^Department of Otorhinolaryngology Head and Neck Surgery, National Academy of Medical Sciences, Kathmandu, Nepal

## Abstract

Hydatid disease is a significant health problem in many livestock-rearing areas especially in the developing world, mainly caused by *Echinococcus granulosus*. The liver and lung are the most common affected sites. However, hydatid disease can occur anywhere in the body. Simultaneous involvement of two organs or sites is very unusual, mainly for organs other than the lung and liver. We thus report a very unusual combination of hepatic and left iliac fossa with hydatid disease in an adult patient. A 37-year-old farmer from a village presented with intermittent right upper quadrant and left iliac fossa pain associated with distention of abdomen for one month. Abdominal radiological investigations reported hydatid cyst disease; one cyst was found in the right lobe of the liver and another in the left iliac fossa. Positive IgG antibody by the ELISA test also confirmed the diagnosis. Pericystectomy and excision of hydatid cyst without spillage of content for the liver and left iliac fossa were done, respectively. Patient was discharged on the 10th postoperative day with an uneventful postoperative course. There was no recurrence of the lesion during one-year follow-up period. A combination of hydatid disease in the liver and iliac fossa is very unusual, so clinician should have thoughts regarding this rare entity as an important differential diagnosis.

## 1. Introduction

Hydatid disease (cystic echinococcosis) is a helminthic infection caused by the larval stages of *Echinococcus*. *Echinococcus granulosus*, *Echinococcus multilocularis*, and *Echinococcus vogeli* or *Echinococcus oligarthrus* are common *echinococci* documented clinically [1, 2]. This disease mainly affects the liver (70%) followed by the lungs (15–47%), kidney (2–4%), bones, and brain. Nevertheless, this can be found anywhere in the body [3]. Simultaneous involvement of two organs occurs in about 5–13% of the cases [4]. However, the left iliac fossa involvement in hydatid disease is unusual and infestation of the left iliac fossa usually occurs by leakage into systemic circulation. It can reach to different parts of the body after absorption from the intestines through the lymphatic routes. It may occur secondary to a ruptured hydatid cyst of the liver or the spleen. Primary left iliac fossa hydatid disease is very uncommon entity accounting for just 2% of all intra-abdominal hydatid disease [5–7].

Thus, hydatid cysts in left iliac fossa may occur as a part of dispersed disease or may occur in isolation. Due to the rarity of left iliac fossa hydatid disease, the possible coexistence of the disease in the liver is more likely. We are reporting infrequent coexistence of hepatic and left iliac fossa hydatid cyst in a young adult patient who was treated successfully without recurrence.

## 2. Case Report

**Figure 1:**
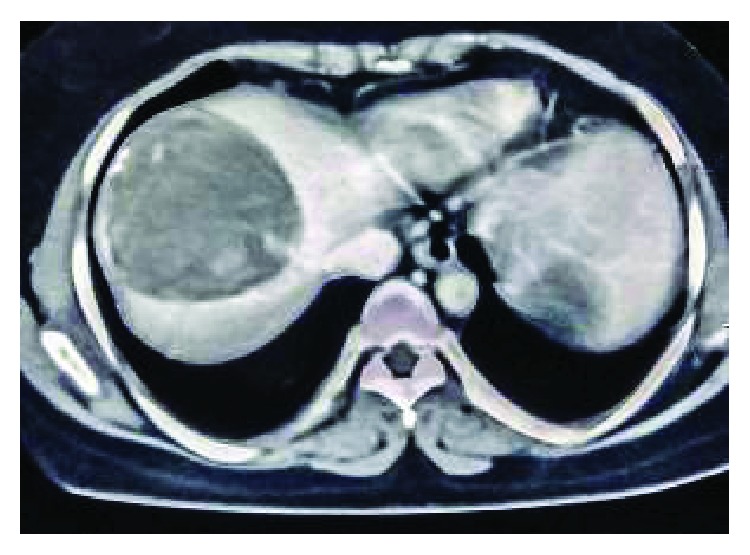
CT scan of abdomen showing giant lobulated hydatid cyst in the right lobe of the liver.

**Figure 2:**
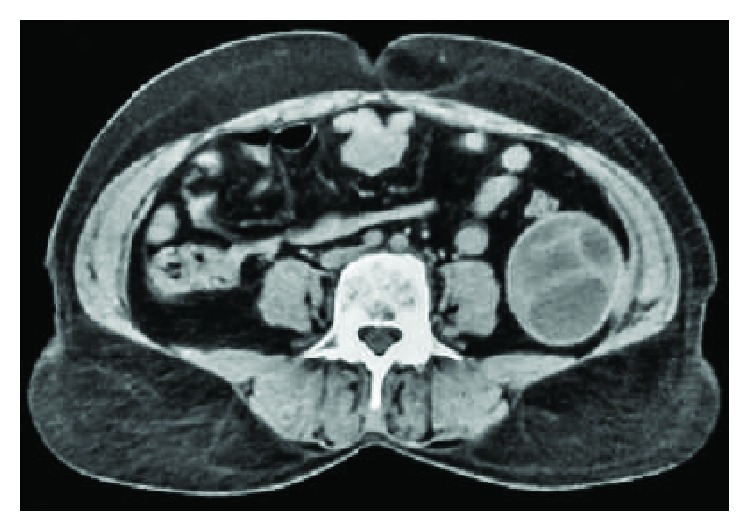
CT scan of the abdomen showing giant multiloculated hydatid cyst with septations in the left iliac fossa.

**Figure 3:**
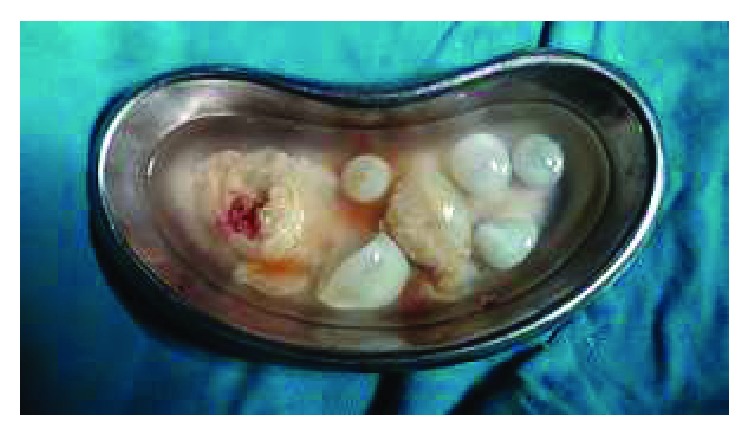
Specimen showing multiple daughter cysts.

A 37-year-old woman presented with twelve-month history of intermittent pain over the right upper quadrant of the abdomen and left iliac fossa. She also complained about distension of the abdomen.There was no history of nausea and vomiting. There was no significant medical, surgical history with normal obstetric history. Her menstrual cycles were normal with last menstrual period one week back. She was a farmer from a poor community with overcrowding and poor hygiene. She had a dog in her house for 5 years. On the examination, her vitals were within normal limits. On the examination of the abdomen, there was tenderness over the right hypochondrium with palpable mass. Similarly, there was nontender smooth palpable mass over left iliac fossa not moving with respiration. Her cardiovascular, respiratory, and neurological examinations revealed no abnormality. Her blood picture was within normal limits except raised eosinophil count (15%). Serological IgG antibody test ELISA for *Echinococcus* was raised (19.5 U/ml). Ultrasonography revealed well-defined hypoechoic heterogenous lesion involving the right lobe of the liver and complex multiloculated anechoic lesion involving left iliac fossa with internal echoes within the lesion suggestive of hydatid disease. CT scan of the abdomen revealed well-defined large complex heterogenous lesion measuring 10 × 8 cm with septations within in 8th lobe of the liver and well-defined lobulated lesion with septation measuring 7 × 6 cm in left iliac fossa suggestive of hydatid cyst (Figures 1 and 2). Intraoperative findings of laparotomy revealed multiple loculated hydatid cysts in the liver and left iliac fossa with multiple daughter cysts (Figure 3). Pericystectomy and excision of hydatid cyst without spillage of content for the liver and left iliac fossa were done, respectively. Postoperative period was uneventful. She was discharged on the 10th postoperative day. She was followed up for twelve months with serial ultrasound abdomen and pelvis scans and clinical examinations.

The patient was treated with albendazole 400 mg two times a day for 3 months to prevent recurrence postoperatively. Patient's blood counts and liver enzymes were monitored during a two-week interval that is given after every month of postoperative albendazole therapy.

## 3. Discussion

Hydatid disease is a significant health problem in many livestock-rearing areas [8]. The increasing trends of people's travel and migration across the world have made it essential for all clinicians to have knowledge about this disease. The prevalence of hydatid diseases ranges from 1 to7%, depending on the endemic area and also the diagnostic test. There are areas where the disease is even higher than 7% [9, 10].

Most of the time, hydatid disease is asymptomatic for a long period of time as the cysts grow very slowly. Cyst may be solitary or multiple. Clinical presentation depends on the number of cysts, size of the cysts, location, and possible compression of adjacent structures [7].

Intra-abdominal hydatid disease may have a vague abdominal mass and pain due to pressure effects on adjacent organs or traction of mesentery. The right upper abdominal or epigastric pain, nausea, vomiting, and hepatomegaly are common symptoms and signs of hepatic hydatid cyst [7]. Cyst rupture or leakage may cause systemic immunological reactions. Our patient had vague right abdominal and left iliac fossa pain due to vague mass in the liver and left iliac fossa. In our case, the left iliac fossa hydatid disease might be due to the rupture of daughter cyst from the main hepatic cyst to the peritoneum. Events as such have been mentioned in the literature [11]. Abdominal trauma and abdominal procedure sometimes may enhance rupture of cyst and may lead to anaphylaxis. Spread of infection retrogradely from the liver through hepatic portal vein into the peritoneal cavity has also been mentioned. Mesenteric cyst, pancreatic cyst, gastrointestinal duplication cyst, ovarian cyst, lymphangioma, intra-abdominal abscess, and hematoma are common differential diagnoses of peritoneal hydatid cyst that should be considered during patient management [12].

Diagnosis of hydatid cyst can be made on the basis of history of exposure, serological tests, and radiological images. Radiological findings may be different according to the stage of the cyst [13]. On the basis of ultrasound imaging findings, World Health Organization (WHO) developed cystic echinococcosis (CE) classification system in 2001 so that same protocol can be followed for the appropriate management of hydatid disease throughout the globe [14]. Computed tomography of the hydatid cysts has a high sensitivity (95–100%). Well-circumscribed, hypodense round lesions without contrast enhancement can be seen in contrast-enhanced CT. Calcification of the cysts can be present in 30% of all cases at the time of diagnosis [15]. Besides radiological methods, serological tests are gaining popularity to confirm a suspected radiologic diagnosis [16].

Complete elimination of the parasite and measures to prevent recurrence should be the goal of management. Risk of mortality due to complications like infections, rupture of cysts and causing anaphylaxis, secondary dissemination, fistulization into the colon, sclerosing cholangitis, and portal hypertension should be born in mind and can be reduced with proper patient management [13]. For small (<5 cm) cysts or for inoperable patients, benzimidazole compounds like mebendazole (MBZ) and albendazole (ABZ) are commonly used [2]. To reduce the risk of anaphylactoid reactions and prevent recurrence, preoperative administration of benzimidazole agents is recommended. Postoperative administration of ABZ for at least 1 month or MBZ for 3 months is also important [13, 17]. Our patient also received preoperative and postoperative albendazole therapy.

For the larger cysts with multiple daughter cysts and hydatidosis, rupture-prone hydatid cysts, and all complicated cysts, surgery (laparoscopic or open) is the treatment of choice [18]. Inactivating infectious material, preventing contamination, eliminating all viable elements (endocyst), and managing the residual cavity remain the main concern during surgical management. The nature of the surgical intervention (pericystectomy, deroofing, capitonnage, hepatectomy, excision) has to be individualized for each patient. Although complete cyst excision with no spillage or cyst rupture is best, it is not every time possible. In those situations, pericystectomy or deroofing or cappittonage with or without omentoplasty is accomplished [19, 20]. Puncture, aspiration, injection, and respiration (PAIR) of a protoscolicidal agent (e.g., hypertonic saline or 95% ethanol) for at least 15 min are alternative methods which have good success rate [21]. In our patient due to the large-sized hepatic lesion and concomitant left iliac fossa lesions, we performed abdominal exploration. Partial cystectomy was done for liver cyst sterilizing with hypertonic saline irrigation. Complete cyst excision was done for left iliac fossa hydatid cyst without spillage of content. Prevention of intraoperative spillage of cyst content to peritoneal cavity is essential since ruptured cysts can cause to anaphylaxis. In case if such events prompt, management of anaphylaxis should be done. Proper identification and management of complicated cysts requires specialized hepatobiliary surgical team [22].

## 4. Conclusions

Concomitant occurrence of hydatid disease in the liver and iliac fossa is very uncommon; thus, a clinician should have also considered it as one of the important differential diagnoses mainly in the developing world where hydatid disease is endemic.
